# Age Difference in Social Discounting: Generous Level Also Makes a Difference

**DOI:** 10.1093/geroni/igaa057.1332

**Published:** 2020-12-16

**Authors:** Hongmei Lin, Xin Zhang

**Affiliations:** Peking University, Beijing, China

## Abstract

Social discounting refers to the phenomenon that individuals’ generous behaviors decline as the social distance increases. But little is known how age could influence social discounting. The present study aimed at a more comprehensive understanding of age-related differences in social discounting. Moreover, as previous studies suggested that older adults are more loss aversion, we would also test whether framing (gain vs loss) could influence social discounting between two age groups. A mixed-model factorial design of 2 (age group: younger vs. older adults) × 2 (framing: gain vs. loss) × 2 (generous level: low vs. high) × 8 (social distance) was conducted, with a total of 78 younger adults and 82 older adults. A significant social distance × age interaction was found, which replicated previous studies suggesting that older adults are more generous toward socially distant others. Interestingly, a significant age × framing × generous interaction was also found, such that in low generous condition, older adults tend to be more generous than younger adults under both gain and loss framing, while such age difference disappeared in high generous condition. These findings indicate that generous level has a positive impact on people social discounting, inducing younger adults to get more generous. Contrary to our expectation, the framing of gain and loss seems not to wave individuals’ social discounting. It seems that people think more seriously about the amount of allocation rather than framing.

